# Unveiling Surface Chemistry of Ultrafast-Sintered LLZO Solid-State Electrolytes for High-Performance Li-Garnet Solid-State Batteries

**DOI:** 10.1021/acs.chemmater.4c02351

**Published:** 2024-11-05

**Authors:** Huanyu Zhang, Matthias Klimpel, Krzysztof Wieczerzak, Romain Dubey, Faruk Okur, Johann Michler, Lars P.H. Jeurgens, Dmitry Chernyshov, Wouter van Beek, Kostiantyn V. Kravchyk, Maksym V. Kovalenko

**Affiliations:** † Laboratory for Thin Films and Photovoltaics, Empa—Swiss Federal Laboratories for Materials Science and Technology, Überlandstrasse 129, CH-8600 Dübendorf, Switzerland; ‡ Laboratory of Inorganic Chemistry, Department of Chemistry and Applied Biosciences, ETH Zürich, Vladimir-Prelog-Weg 1, CH-8093 Zürich, Switzerland; § Laboratory for Mechanics of Materials and Nanostructures, Empa—Swiss Federal Laboratories for Materials Science and Technology, Feuerwerkerstrasse 39, CH-3602, CH-8093 Zürich, Switzerland; ∥ Laboratory for Joining Technologies & Corrosion, Empa—Swiss Federal Laboratories for Materials Science and Technology, Überlandstrasse 129, CH-8600 Dübendorf, Switzerland; ⊥ Swiss-Norwegian Beamlines, European Synchrotron Radiation Facility, 71 Av. des Martyrs, 38000 Grenoble, France

## Abstract

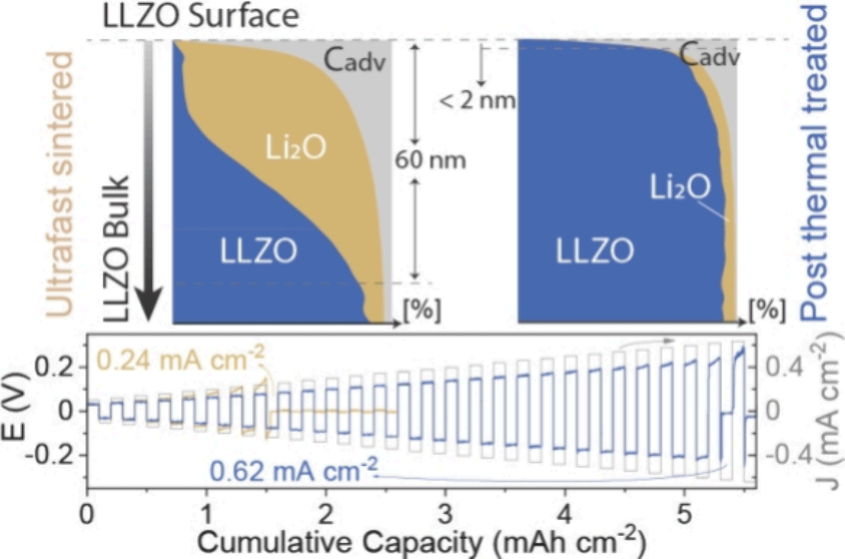

Ultrafast (UF) sintering emerges as a game-changing sintering methodology for fabricating Li_7_La_3_Zr_2_O_12_ (LLZO) solid-state electrolytes, representing a pivotal stride toward the advancement and prospective commercialization of Li-garnet solid-state batteries. Despite its widespread use in the fabrication of LLZO ceramics, the chemical composition of the UF-sintered LLZO surface remains largely unexplored. This study presents an in-depth analysis of the surface chemistry of UF-sintered LLZO using comprehensive techniques, including depth-profiling X-ray photoelectron spectroscopy (XPS) and focused-ion-beam time-of-flight secondary ion mass spectroscopy (FIB-TOF-SIMS). Our investigation uncovers a striking difference between the surface of UF-sintered and conventionally sintered LLZO, revealing predominant surface contamination by Li_2_O up to ca. 40 nm depth in the case of UF processing. Comparative synchrotron X-ray diffraction data during UF and conventional sintering elucidate the origin of surface contamination. We propose a viable solution to this issue through an additional heat treatment (HT) step at 900 °C after UF sintering, as corroborated by XPS and FIB-TOF-SIMS measurements. Furthermore, we present a comparative assessment of the electrochemical performance of Li/LLZO/Li symmetric cells based on UF-sintered LLZO pellets, both with and without the post-HT step, underscoring the pivotal role of an uncontaminated LLZO surface.

## Introduction

The current battery research landscape is witnessing a significant shift toward substituting liquid electrolytes in Li-ion batteries with safer, nonflammable solid alternatives, particularly focusing on Li_7_La_3_Zr_2_O_12_ (LLZO) with garnet-type structure.^1−3^ LLZO solid-state systems hold promise not only in addressing the pressing need for safe and temperature-tolerant batteries but also in enhancing their energy density and cycle life compared to traditional liquid-electrolyte-based counterparts.^4−8^ Such prospects stem from intrinsic properties of LLZO including its superior thermal stability, high Li-ion conductivity of up to 1 mS cm^–1^ at room temperature (RT),^3,9,10^ relatively low electronic conductivity of ca. 10^–8^ S cm^–1^ at RT,^11^ and chemical stability in contact with metallic Li. However, integrating LLZO solid-state electrolytes (SSEs) into batteries has encountered substantial challenges, particularly in sintering processes.^12^ Major issues revolve around maintaining the proper Li stoichiometry (cubic LLZO structure, c-LLZO) after sintering, ensuring reproducibility of the LLZO microstructure, and addressing cost and scalability concerns associated with the sintering process. In response, recent efforts have focused on innovative sintering technologies for LLZO ceramics, including microwave-assisted sintering,^13,14^ spark plasma sintering,^15^ flash sintering,^16^ and ultrafast (UF) sintering.^17−19^

Among these methods, UF sintering emerges as a frontrunner due to its potential to reduce sintering duration of LLZO ceramics from hours to seconds, enabling precise control over Li stoichiometry and the LLZO microstructure. It is considered a scalable roll-to-roll sintering process, potentially streamlining the commercialization of Li-garnet solid-state batteries. Unlike conventional sintering methods, where the ceramic sample is positioned relatively far from the heating elements, limiting the heating rate, UF sintered sample is placed much closer to the heat source. The rapid thermal shock promotes particle fusion, significantly reducing both the sintering time and temperature required for LLZO ceramics. Consequently, lithium losses are minimized, which helps prevent the formation of the poorly conductive La_2_Zr_2_O_7_ secondary phase. Notably, UF sintering gained significant attention following the publication of Hu and co-workers^17^ in 2020, demonstrating the sintering of LLZO pellets between two joule-heated carbon felts. Subsequent studies further expanded UFS applicability,^18−26^ including the fabrication of thin LLZO membranes.^27−29^

Despite the widespread use of UF sintering for the fabrication of LLZO ceramics, comprehensive studies of the LLZO surface after UF sintering are surprisingly scarce. This research gap is critical considering that the presence of secondary phases such as Li_2_O and La_2_Zr_2_O_7_ on the LLZO surface is poised to detrimentally impact the electrochemical performance of the Li anode when coupled with UF-sintered LLZO. Their notably lower Li-ion conductivities compared to crystalline cubic phase LLZO (c-LLZO) could not only increase the voltage polarization of the full cell, but also lead to uneven Li electrodeposition at the LLZO/Li interface, thereby initiating the formation of Li dendrites.

This study focuses on elucidating the chemical composition of LLZO surface after UF-sintering, employing a multifaceted approach encompassing X-ray photoelectron spectroscopy (XPS) coupled with Ar sputtering and focused ion beam time-of-flight secondary ion mass spectroscopy (FIB-TOF-SIMS). Our investigation reveals that the surface of UF-sintered LLZO is predominantly composed of Li_2_O up to a depth of ca. 40 nm, indicating a significant difference in chemical composition compared to the surface of conventionally sintered LLZO. Furthermore, synchrotron X-ray diffraction (SXRD) measurements performed during UF and conventional (slow) sintering shed a light on the origin of this surface contamination. In addition, we elucidate the impact of additional heat-treatment (HT) of the LLZO surface after UF sintering. Specifically, we show that a 10-min-post-HT step at 900 °C effectively eliminates Li_2_O contamination, as confirmed by XPS, and FIB-TOF-SIMS measurements. The comparisons of the electrochemical performance of Li/LLZO/Li symmetric cells based on UF-sintered LLZO pellets, with and without the post-HT step, underscored the importance of a non-Li_2_O contaminated LLZO surface.

## Results and Discussion

**Figure 1:**
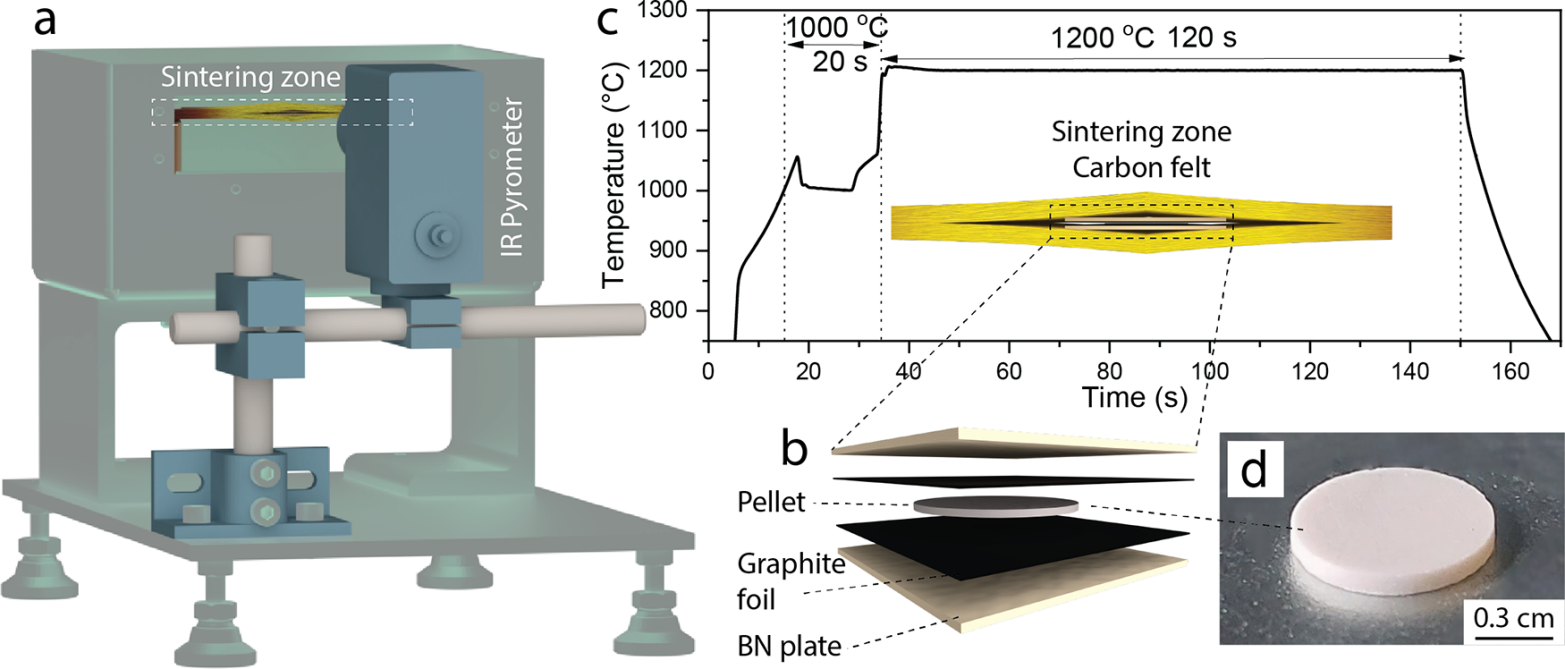
(a) Schematic of the UFS setup. (b) Detailed configuration of the UFS heating zone. (c) Typical temperature profile of UFS. (d) Photograph of the UF-sintered LLZO pellet.

UF sintering of as-prepared (green body) LLZO pellets was performed in an Ar-filled glovebox using a custom-made UF sintering setup (Figures 1a and S1), containing two superimposed carbon felts clamped between two copper electrodes. To remove moisture and Li_2_CO_3_ from the LLZO surface of green body pellets prior to sintering, the pellets were dried at 200 °C for 30 min, followed by a heat-treatment at 900 °C for 10 min in an Ar-filled glovebox. In our UFS experiments, we used a typical sintering configuration where the LLZO pellets were positioned between two carbon foils (Figures 1b and S1), and then sandwiched between two BN plates and two stretched carbon felts.^27^ The BN substrate was selected due to its high thermal conductivity of ca. 550 W m^–1^ K^–1^, thermal stability within the operating temperature range, and superior structural resistance to thermal shock.^30,31^ The UF sintering setup was powered by a DC source and the temperature of the sintering zone was monitored by an infrared camera. As-prepared, pressed LLZO pellets were first heated to 1000 °C for 20 s to stabilize the temperature therefore to minimize overshoot. The actual sintering was then conducted at 1200 °C for 120 s to obtain mechanically robust and well-sintered ceramic pellets (Figure 1d). A typical UF sintering temperature profile is shown in Figure 1c. Importantly, as shown in Figure S2a,b, powder X-ray diffraction (PXRD) and Raman spectroscopy measurements confirmed the pure cubic structure of the LLZO pellets after UF sintering (space group *Ia*3̅*d*, *a* = 12.9622(2) Å, *V* = 2177.89 Å^3^, ICSD 235896). Scanning electron microscopy (SEM) images of LLZO pellets before and after sintering (Figure S3a,b) show the effectiveness of UFS in producing high-density (91%) LLZO pellets in a short 2 min sintering time. Notably, as shown in Figure S3c, compression tests performed on the UF-sintered LLZO pellets indicated their ability to withstand forces of at least 500 N, which corresponds to a pressure of *ca*. 11 MPa. Such high mechanical stability is a key feature of solid-state electrolytes when they are used in solid-state batteries. The Li-ion conductivity and activation energy of the UF-sintered LLZO pellets were estimated at the level of 0.19 mS cm^–1^ (at RT) and 0.39 eV, respectively (Figure S2c,d). These values align closely with previously reported data for LLZO pellets.^9,10^

To assess the chemical purity of the surface of UF-sintered LLZO pellets, they were analyzed in depth by XPS combined with Ar-ion sputtering depth profiling. Ar-ion sputtering was performed in multiple 1 min steps, each corresponding to a sputtering depth of ca. 2 nm. XPS measurements were limited to the specific binding energy ranges corresponding to O 1s, C 1s, Zr 3d, La 3d, and Li 1s spectra, as no additional elements were identified from the analysis of the XPS survey spectra of an UF-sintered LLZO pellet (Figure S5). It should be noted that the measured LLZO samples were not exposed to air during transfer to the XPS system as it was directly connected to the Ar-filled glovebox.

**Figure 2:**
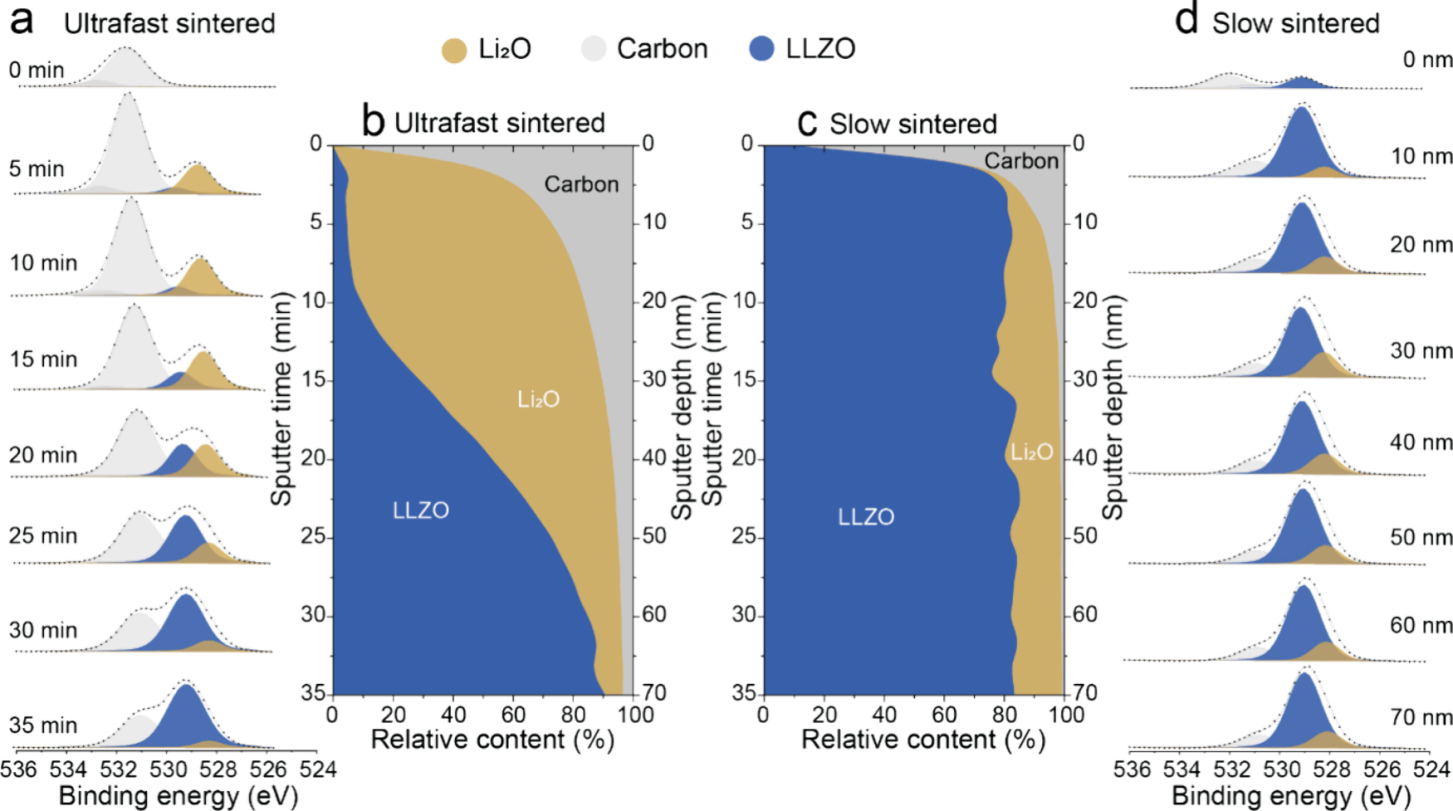
Charge-corrected O 1s spectra of UF (a) and slowly (d) sintered LLZO pellets collected on the as-sintered LLZO surface and after its sputtering for 0–35 min (sputtering depth of 0–70 nm). Chemical composition of UF (b) and slowly (c) sintered LLZO pellets as a function of the sputtering depth. La 3d, C 1s, Zr 3d, Li 1s, Zr 4s are shown in Figures S4 and S7, respectively.

As shown in Figure 2a, the surface of LLZO consists mainly of adventitious carbon, as evidenced by the presence of the C 1s peaks at 284.8, 286.1, 289.1 eV and the O 1s peaks at 532.7, 531.5 eV, which can be unambiguously assigned to C–C, C=O, O=C–O, and O=C, O=C–O bonds, respectively. The La 3d, Zr 4s, and O 1s peaks at 833.7–838.4 eV, 181.2–183.6 and 529.5 eV are clearly assigned to LLZO. Another O 1s peak at 528.6 eV in the sputtering depth range of 10–70 nm can be attributed to Li_2_O (Li–O bonds).^32^ To estimate the relative composition of the LLZO surface as a function of sputtering depth, the sum of the intensities of each O 1s peak was equated to 100%, and thus the relative fraction of each O 1s peak corresponding to adventitious carbon, LLZO, or Li_2_O was calculated (see Experimental section for details). The results are summarized in Figures 2b and S6 in the form of compositional sputtering depth profiles and chemical composition-sputtering depth 2D map.

According to the findings depicted in Figure 2b, the surface composition of LLZO predominantly consists of adventitious carbon, attributed to the interaction between volatile organics in the glovebox and the LLZO surface. Within the sputtering depth range of 10–40 nm, the LLZO surface comprises primarily of Li_2_O, with minor quantities of LLZO and adventitious carbon. At a sputtering depth of 60–70 nm, the surface is primarily composed of LLZO structure. Importantly, our reference XPS experiments, conducted on LLZO pellets sintered in a muffle furnace (located in the same glovebox next to the UFS setup) employing a slower heating/cooling rate of ca. 20 K min^–1^ and a dwell time of 30 min, revealed that their surface is primarily composed of LLZO lattice with small content of Li_2_O (Figures 2c,d, S6, and S7).

**Figure 3:**
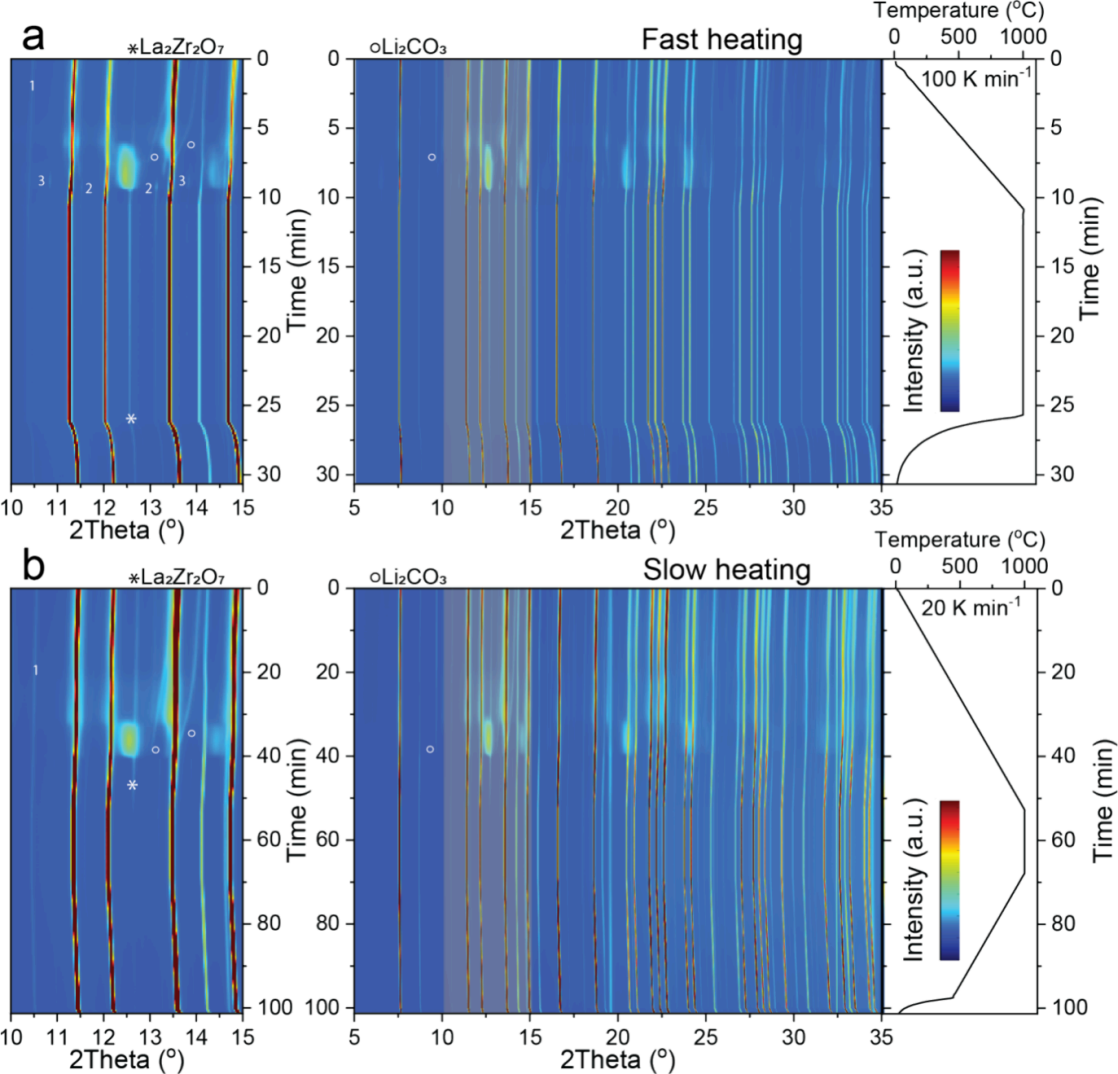
(a, b) Combined SXRD patterns of LLZO powder measured during fast (a, 100 K min^–1^) and slow (b, 20 K min^–1^) heating to 1000 °C, held at 1000 °C for 15 min, followed by fast (ca. 100 K min^–1^) and slow (20 K min^–1^) cooling. Left side: enlarged SXRD regions between 10 and 15 2Theta (λ = 0.68966 Å). Right side: employed temperature profiles during operando SXRD. SXRD reflections of LaAlO_3_, La_2_O_3_, and ZrO_2_ secondary phases are denoted as 1, 2, and 3, respectively.

For comprehending the reasons of the presence of Li_2_O in relatively large quantities at the surface of the UF-sintered LLZO pellets, in situ SXRD measurements were conducted during the heat-treatment of LLZO powder up to 1000 °C (Figure 3a) at a heating ramp of ca. 100 K min^–1^. Notably, it was not possible to perform measurements up to 1200 °C and at higher heating ramp, due to the limitations of the setup used. Analysis of the XRD patterns revealed several significant observations. First, as follows from the XRD pattern of the LLZO powder before sintering, it contains small quantities of the La_2_Zr_2_O_7_ and Li_2_CO_3_ impurities (Figure S8a), the presence of which was additionally confirmed by Raman spectroscopy measurements on the pristine LLZO powder (Figure S11). As consistent with previous research,^33−38^ the occurrence of La_2_Zr_2_O_7_ and Li_2_CO_3_ phases can be attributed to the interaction of the LLZO surface with H_2_O and CO_2_. Specifically, as was revealed by Larraz et al.^39^ and Vema et al.,^40^ LLZO first reacts with water to form LiOH, and then further reacts with CO_2_ to form La_2_Zr_2_O_7_, La_2_O_2_CO_3_, and Li_2_CO_3_. Consequently, both La_2_Zr_2_O_7_ and Li_2_CO_3_ secondary phases, as identifies through SXRD, along with presumably amorphous LiOH, accumulate on the LLZO surface during handling and storage of LLZO powder in air. It is important to highlight that previous research^39−41^ has shown that besides LiOH and Li_2_CO_3_, the reaction of LLZO with water and CO_2_ yields several additional products. These include La_2_(OH)_2_(CO_3_)_2_, La_2_Zr_2_O_7_, and protonated LLZO (h-LLZO). Second, upon heating of LLZO samples, the decrease and disappearance of the intensities of Li_2_CO_3_ reflections was observed as a result of Li_2_CO_3_ melting and decomposition leading to the formation of amorphous Li_2_O and CO_2_ gas.^42^ This process was followed by the rapid increase and then decrease in intensity and broadening of La_2_Zr_2_O_7_ reflections in the very narrow temperature range of 625–770 °C, accompanied by the concomitant appearance of ZrO_2_ and La_2_O_3_ secondary phases. The increase in the intensity of the La_2_Zr_2_O_7_ phase and the broadening of its reflection, indicating the formation of nanostructured La_2_Zr_2_O_7_ at ca. 625 °C, can be elucidated by a rapid shift in the equilibrium of the thermal decomposition reaction of Li_2_CO_3_. Specifically, it is conceivable that during the thermal decomposition of Li_2_CO_3_, the partial pressure of CO_2_ within the contracting pores of the densifying LLZO pellet rises significantly, shifting the equilibrium to favor the formation of amorphous Li_2_CO_3_. At this transient stage, it can be hypothesized that CO_2_ reacts not only with Li_2_O but also interacts with the LLZO surface within the pores, thus triggering the formation of the nanostructured La_2_Zr_2_O_7_ phase and molten Li_2_CO_3_. However, this process is fleeting, lasting merely two minutes, following which the equilibrium reverts to favor the decomposition of Li_2_CO_3_ into Li_2_O and CO_2_. Subsequently, this leads to a simultaneous chemical reaction between Li_2_O and the newly formed nanostructured La_2_Zr_2_O_7_, resulting in the formation of cubic LLZO phase.

The examination of XRD data from 625 °C onward implies that while the intensity of the La_2_Zr_2_O_7_ reflections gradually diminishes, pointing to the ongoing solid-state reaction of the La_2_Zr_2_O_7_ phase with Li_2_O toward the formation of cubic LLZO, traces of La_2_Zr_2_O_7_ are still present in the LLZO pellet even after the completion of the UF sintering process. These findings, coupled with XPS data comparing UF- and conventionally sintered LLZO pellets, indicate that the abundance of Li_2_O on the surface of ultrafast sintered LLZO pellets primarily originates from the presence of Li_2_CO_3_ on the LLZO particle surface, which decomposes to Li_2_O and CO_2_ during the heat-treatment of raw LLZO powder prior to UF sintering. Conversely, the small quantity of Li_2_O on the surface of conventionally sintered pellets is evidently linked to the notably longer sintering duration. This prolonged time at high temperatures enables the completion of the reaction between La_2_Zr_2_O_7_ and Li_2_O, ultimately leading to the formation of the c-LLZO phase. This assumption was confirmed by SXRD results performed on as-pressed LLZO during their sintering at slow heating rate of 20 K min^–1^, indicating a complete disappearance of the La_2_Zr_2_O_7_ phase after sintering (Figures 3b and S8b).

**Figure 4:**
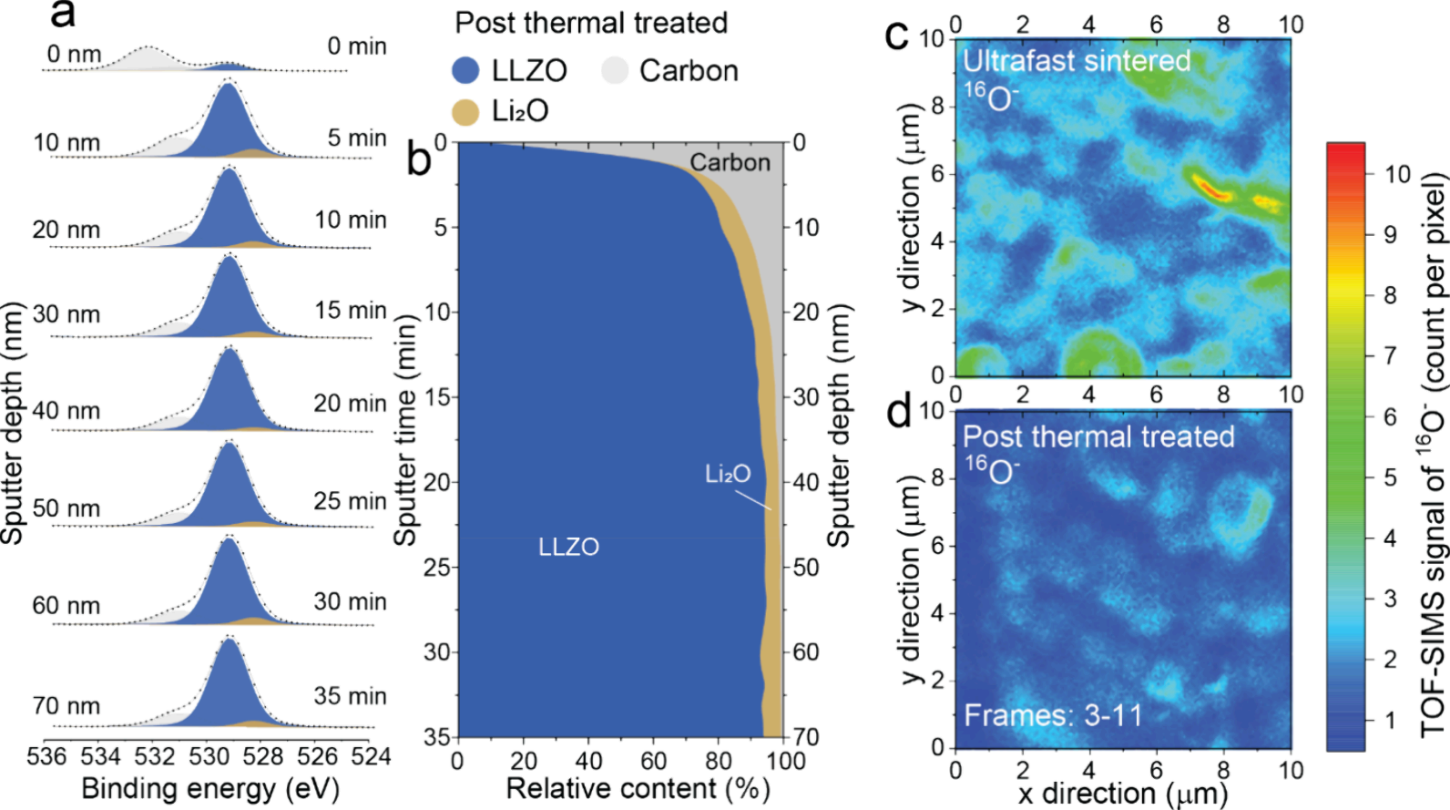
(a) Charge-corrected O 1s spectra and (b) chemical composition of UF-sintered LLZO pellets after additional 900 °C (10 min) heat-treatment step at different sputtering depths of 0–70 nm. (c, d) TOF-SIMS 2D maps of oxygen distribution (^16^O^–^ signal) in UF-sintered LLZO pellets without (c) and with (d) additional heat-treatment step at 900 °C (10 min), measured over the LLZO surface area of 100 μm^2^ and the thickness of ca. 400 nm (from 100 to 500 nm from the surface).

Subsequently, in an effort to remove the Li_2_O-contamination layer from the surface of UF-sintered LLZO pellets, we conducted an additional heat-treatment at 900 °C for 10 min, followed by comprehensive surface characterization using XPS combined with Ar-ion sputter depth profiling. The findings are summarized in Figure 4a,b, demonstrating both the O 1s spectra and the chemical composition-sputtering depth map of the surface after the 900 °C heat-treatment. Figures S5, S6, and S9 illustrate the measured XPS survey spectra, La 3d, C 1s, Zr 3d, Li 1s, and Zr 4s spectra at specific sputtering depths, along with the corresponding compositional sputter-depth profiles. Similar to the XPS data obtained from UF-sintered LLZO pellets without heat-treatment, the O 1s peaks at 529.3, 528.4, 531.4, and 532.5 eV were assigned to LLZO, Li_2_O, and adventitious carbon, respectively. Importantly, a direct comparison between the XPS data acquired from UF-sintered LLZO pellets without (Figure 2a,b) and with (Figure 4a,b) additional heat-treatment clearly indicates that the heat-treatment effectively reduces the amount of Li_2_O on the LLZO surface. This reduction results from the solid-state reaction between La_2_Zr_2_O_7_ and Li_2_O, a process that was not fully completed during UF sintering.

Next, to reveal the distribution of Li_2_O within the UF-sintered LLZO pellets before and after an additional postheat treatment step, we conducted a 2D mapping of oxygen distribution (TOF-SIMS signal of ^16^O^–^) over the LLZO surface area of 100 μm^2^ (10 μm × 10 μm) and at a thickness of ca. 400 nm (from 100 to 500 nm from the surface). The mapping was performed using fluorine gas-assisted FIB-TOF-SIMS, as illustrated in Figures 4c,d and S10. This innovative technique, employing fluorine gas assistance, significantly enhances the sensitivity of the SIMS signal. The ^16^O^–^ distribution was obtained by integrating 9 frames (frames 3–11) during a sputtering period lasting ca. 287 s. The first 2 frames were excluded from the analysis due to the presence of a protective gold layer on the LLZO surface. Given that the density of oxygen atoms in Li_2_O is about 10 times higher than in LLZO, an increased intensity of the ^16^O^–^ signal can indicate a higher concentration of Li_2_O within a specific spatial region. Comparing the TOF-SIMS 2D maps of UF-sintered LLZO pellets before and after the additional heat-treatment at 900 °C, it can be inferred that Li_2_O in UF-sintered LLZO pellets is likely present in grain boundaries. After the heat treatment step, Li_2_O disappears, confirming the previously discussed assumption on the required completion of the solid-state reaction of La_2_Zr_2_O_7_ with Li_2_O after UF sintering.

**Figure 5:**
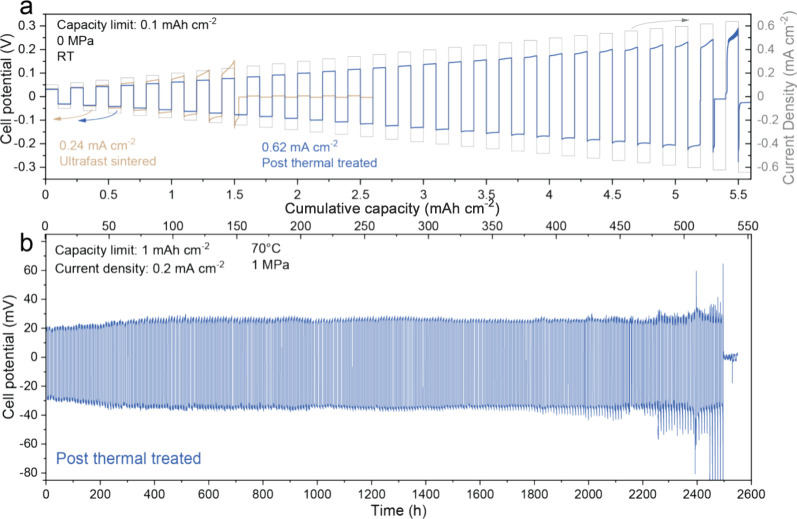
(a) Voltage profiles of the Li/LLZO/Li symmetrical cells, assembled with UF-sintered LLZO pellets with and without additional 900 °C-heat-treatment step, measured at different current densities ranging from 0.1 mA cm^–2^ to 0.64 mA cm^–2^ with an increasing step of 0.02 mA cm^–2^ and a capacity limitation of 0.1 mA h cm^–2^ per half-cycle. The measurements are conducted without external pressure at room temperature. (b) Voltage profiles of the Li/LLZO/Li symmetric cell, assembled with UF-sintered LLZO pellets with additional 900 °C heat-treatment step, measured at a current density of 0.2 mA cm^–2^, the areal capacity limitation of 1 mA h cm^–2^ per half-cycle at 70 °C.

To evaluate the electrochemical performance of UF-sintered LLZO pellets after heat-treatment with respect to Li plating/stripping, we conducted critical current density (CCD) measurements using symmetrical Li/LLZO/Li cells (Figure 5a). The CCD, denoting the current density initiating Li dendrite propagation, was determined through galvanostatic cycling experiments at various current densities.^43^ Specifically, the current density was increased in steps from 0.1 to 0.64 mA cm^–2^ in increments of 0.2 mA cm^–2^, carrying 0.1 mA h cm^–2^ of Li for each half cycle. Fabrication of the Li/LLZO/Li symmetrical cells involved isostatic pressing of lithium foil ca. 100 μm thick, at around 350 MPa onto 800 μm-thick LLZO pellets. As shown in Figure 5a, a distinct voltage drop occurred during the 27th half-cycle of the Li/LLZO/Li symmetrical cell, revealing its CCD of 0.62 mA cm^–2^. Notably, this CCD value closely resembles those reported for conventionally sintered LLZO pellets using a muffle furnace.^44^ In contrast, analogous measurements conducted on a symmetric cell based on UF-sintered LLZO pellets without heat-treatment resulted in a short circuit at the eighth half-cycle, corresponding to a CCD of 0.24 mA cm^–2^. This notably low CCD value is primarily attributed to void formation at the Li/LLZO interface during Li stripping, evident from the curved voltage profiles, particularly noticeable after the fifth cycle, indicating deviation from Ohmic behavior. Consequently, it can be inferred that the presence of Li_2_O on the LLZO pellet surface not only increases the Li/LLZO interfacial resistance but also intensifies void formation.^45,46^ This leads to a reduced contact area between Li and LLZO, resulting in localized higher current densities known as the current focusing effect at the Li/LLZO interface during subsequent Li plating. The latter promotes Li dendrite growth at significantly lower current densities than those required for dendrite formation in an unstripped Li/LLZO interface. Importantly, additional galvanostatic measurements of Li/LLZO/Li symmetric cells assembled with heat-treated LLZO pellets and operated at a current density of 0.2 mA cm^–2^ with a limiting areal capacity of 1 mAh cm^–2^ per half cycle have demonstrated superior cycling stability. As shown in Figure 5b, the cells were stable for 2500 h, corresponding to ≈525 mA h cm^–2^ of cumulative Li areal capacity, and exhibited a constant overpotential of ≈20 mV throughout the cycling measurements. These data obtained on US-sintered LLZO pellets are comparable to the electrochemical performance measured on conventionally sintered LLZO pellets.^44,47,48^

## Conclusions

This study highlights a previously overlooked yet critical issue associated with UF sintering of LLZO solid-state electrolytes–Li_2_O contamination on the LLZO surface. Detailed depth-profiling XPS characterization of UF-sintered LLZO has unveiled a predominant presence of Li_2_O up to ca. 40 nm in thickness on the LLZO surface, a phenomenon notably less pronounced in conventionally sintered LLZO pellets. Using SXRD during both UF and slow sintering processes, we have revealed that this striking difference in surface composition stems from an incomplete solid-state reaction occurring between Li_2_O and La_2_Zr_2_O_7_ during UF sintering. Notably, the presence of La_2_Zr_2_O_7_ is associated with the interaction of LLZO with H_2_O and CO_2_ during LLZO storage and handling, resulting in the formation of La_2_Zr_2_O_7_ and Li_2_CO_3_. Li_2_O forms from the thermal decomposition of Li_2_CO_3_ during sintering.

Moreover, our investigations into the impact of additional HT of the LLZO surface after UF sintering have revealed its efficacy in completing the Li_2_O/La_2_Zr_2_O_7_ solid-state reaction (at *T* > 600 °C), as validated by XPS and FIB-TOF-SIMS analyses. Importantly, Li/LLZO/Li symmetric cells assembled with UF-sintered LLZO pellets following the 900 °C-HT step exhibited a considerable enhancement, demonstrating a superior CCD of up to 0.6 mA cm^–2^ and reduced voltage polarization compared to non-HT UF-sintered LLZO pellets. Noteworthy, the cyclic assessment of Li/LLZO/Li symmetric cells based on UF-sintered LLZO pellets with a 900 °C-HT step revealed their superior stability, sustaining over 550 cycles at a current density of 0.2 mA cm^–2^ while maintaining an areal capacity limit of 1 mA h cm^–2^. These results underscore the critical importance of eliminating Li_2_O residues on the LLZO surface after UF sintering, opening a pathway to robust, high-performance Li-garnet solid-state batteries.

## Experimental Section

### Preparation of LLZO Pellets

240 mg of aluminum-doped LLZO (Al-LLZO powder from Ampcera) was loaded into a pressing die (*d* = 1 cm). The powder was then uniaxially compressed with a force of ca. 10 kN. After compression, the surface of the green body pellets was carefully polished using SiC abrasive paper to remove any visible impurities. Subsequently, to remove moisture and Li_2_CO_3_ from the LLZO surface, the pellets were dried on an alumina plate at 200 °C for 30 min in air, followed by a heat-treatment at 900 °C for 10 min in an Ar-filled glovebox (Inert Corp, O_2_ < 0.1 ppm, H_2_O < 0.5 ppm) using a sacrificial LLZO pellet as a substrate. Comparing the Raman spectra of the raw LLZO powder with the as-prepared green body LLZO pellet before UF sintering (Figure S11), it is evident that this heat-treatment effectively eliminates Li_2_CO_3_. UF sintering of the prepared green body LLZO pellets was performed in an Ar-filled glovebox using a custom setup, as described in ref (27). The pellets were first preheated to 1000 °C for 20 s and then sintered at 1200 °C for 120 s. The heating and cooling rates were ca. 100 °C s^–1^ (see Figure 1c for a typical temperature profile). The pellets were sandwiched between BN substrates as a mechanical support to prevent LLZO pellets bending. BN substrates were selected due to high thermal stability, thermal conductivity and low electronic conductivity. Graphite foil were also applied to be as a chemical block between LLZO and BN substrates. The UFS setup was operated with and DC power source (Aim-TTi CPX400DP Dual 420 W PowerFlex DC Power supply). The sintering temperature was monitored by an IR camera (MAURER Pyrometer KTRD 4085-1). In the case of performing the post-treatment step, the UFS sintered LLZO pellets were transferred into the muffle furnace (located in the same Ar-filled glovebox as UFS setup), placed on a sacrificial LLZO substrate and then heat-treated at 900 °C for 10 min (the heating and cooling rates were ca. 50 and 10 °C min^–1^, respectively). Importantly, to verify the preservation of the cubic LLZO phase, XRD measurements on UF-sintered LLZO pellets following a 900 °C post-treatment were performed (Figure S12). The XRD data confirmed that the 10-min heat-treatment duration is sufficiently short to prevent the decomposition of the cubic LLZO phase.

### Material Characterization

PXRD was measured on a STOE STADI P powder X-ray diffractometer in transmission mode (Cu-K_α1_ irradiation, λ = 1.5406 Å). Cross-section SEM images were recorded on a Hitachi TM3030Plus tabletop microscope with an acceleration voltage of 5 kV. Raman spectroscopy measurements were performed using a Horiba LabRAM HR Evolution UV–vis–NIR system equipped with a 532 nm laser operating at a power of 300 mW. To prevent exposure of the samples to air, they were carefully sealed between two thin glass slides in an Ar-filled glovebox. The compression test of ultrafast sintered LLZO pellets was performed using a Tinius Olsen ST1 universal testing machine equipped with a 500 N load sensor, at a constant piston speed of the of 2.5 μm s^–1^ and a preload force of 1 N.

In situ synchrotron XRD measurements were performed at the Swiss Norwegian Beamline (SNBL) BM01 at the European Synchrotron Radiation Facility (ESRF, Grenoble, France) using the PILATUS@SNBL diffractometer (λ = 0.68966 Å) in a high-intensity beam-mode (200 mA). The experiments were conducted in a custom-made furnace (see ref (49)) under nitrogen atmosphere. The samples were heated to 1000 °C for 15 min using fast (100 K s^–1^) and slow (5 K s^–1^) heating rates. The data acquisition time was 10 s per pattern. The 2D diffraction data from a Pilatus 2 M detector were processed using the SNBL Toolbox and BUBBLE software. The visualization of SXRD peaks during sintering was done by Modulation-Enhanced Diffraction Viewer and Editor (Medved) software, developed by SNBL (ref (50)).

XPS analysis was performed using a PHI Quantes spectrometer (ULVAC-PHI), equipped with a conventional low-energy Al-Kα source (1486.6 eV). The XPS spectrometer was directly connected to an Ar-filled glovebox, thus enabling an in situ transfer of LLZO pellets from the glovebox of the UFS setup to the XPS analysis chamber without intermediate exposure to air. The energy scale of the hemispherical analyzer was calibrated according to ISO 15472 standards. This calibration process involved referencing the Au 4f7/2 and Cu 2p3/2 primary peaks (measured in situ with corresponding sputter-cleaned, high-purity metal references) to the recommended binding energy (BE) positions of 83.96 and 932.62 eV, respectively. Additionally, to maintain charge neutrality during each measurement cycle, a dual beam charge neutralization system was employed. This system utilized low-energy electron and argon-ion beams with a bias of 1 V and a current of 20 μA. Compositional sputter-depth profiles were obtained by performing alternating cycles of XPS analysis (Al-Kα at 51 W; beam diameter approximately 200 μm) and sputtering with a focused 1 keV Ar beam, rastering an area of 2 × 2 mm^2^. During each measurement cycle, the Li 1s, La 3d, Zr 3d, C 1s, and O 1s regions were recorded with a step size of 0.05 eV and a pass energy of 280 eV. The etch rate was calibrated to be 2 nm min^–1^ on a 100 nm Ta_2_O_5_/Ta reference sample. XPS survey spectra, covering the binding energy (BE) range of 0 eV–1200 eV (XPS), were recorded with a step size of 0.5 eV at a constant pass energy of 280 eV using the Al-Kα source (power 51 W; beam diameter ca. 200 μm). The calculated probing depths for the La 3d, Zr 3d, O 1s, C 1s, and Li 1s photoelectron lines, as recorded from LLZO using Al-Kα X-ray radiation (*h*ν = 1486.7 eV) are as follows: La 3d (3.6 nm), Zr 3d (5.5 nm), O 1s (4.8 nm), C 1s (5.5 nm), and Li 1s (6.6 nm).^51^ Charge correction of XPS spectra recorded on UF-sintered LLZO pellets, both before and after Ar sputtering, was performed by referencing the C 1s peak (C–C bond) at 284.8 eV. Specifically, the recorded XPS spectra were shifted according to the difference between the observed adventitious carbon C 1s peak and the reference C 1s peak at 284.8 eV. The same method was used for charge correction of XPS spectra measured on slowly sintered and post-treated LLZO pellets prior to sputtering. However, the charge correction of XPS spectra on slowly sintered LLZO and post-treated LLZO pellets after argon sputtering (at depths of 2–70 nm) was different. The spectra were charge-corrected by aligning the Zr 3d peaks, which are reliably associated with LLZO, to their corrected positions at 0 nm sputter depth. This change in charge correction method was necessary due to the significantly lower intensity of the C 1s peaks at deeper sputter depths, resulting from the reduced presence of adventitious carbon impurities in the slowly sintered and post-treated LLZO samples. Spectral reconstruction was performed by linear-least-squares fitting of the background-corrected spectra with one or more symmetric, mixed Gaussian–Lorentzian line shape functions using the CasaXPS software. The Gaussian fraction of each peak component (representative of instrumental broadening) was constrained to 0.5. The full width at half-maximum (fwhm) and relative BE position of each fitted peak component were kept constant when fitting the peaks at different sputtering depths. The relative intensity of each O 1s peak was calculated from its peak area, normalized to the area of the O 1s peak collected at the last sputter step (sputter time of 35 min), as assigned to the bulk LLZO structure. The binding energy value in this study has a general deviation of ±0.1–0.2 eV, according to instruments error.

TOF-SIMS measurements were performed using FIB/SEM dual-beam system LYRA3 from Tescan (Brno, the Czech Republic) equipped with a high-resolution TOF-SIMS (HTOF) detector from TOFWERK (Thun, Switzerland) and a 5-line gas injection system (GIS) from Orsay Physics (Fuveau, France). The surface of the measured samples was bombarded with a primary ^69^Ga^+^ beam at 30 kV and an ion current of ∼1 nA. The data were acquired with a 30 μs dwell time per pixel. A pixel number of 1024 × 1024 and binning of 4 × 4 pixels were used. The TOF-SIMS data were collected and analyzed using TOF-SIMS Explorer version 2.5.5 (from TOFWERK, Thun, Switzerland). The mass calibration was carried out using the secondary ion signal of the analyzing primary ion beam isotope (^69^Ga^+^) and the most dominant isotope signals. Experiments were performed with coinjection of fluorine during sputtering to increase the ionization probability and to evaluate the effect of topography on the SIMS signal.^52^ Fluorine was obtained by the fragmentation of a XeF_2_ precursor on the sample surface during Ga^+^ beam bombardment. GIS systems are typically used for gas-assisted deposition and etching with focused electron or ion beam (FEB/FIB). To adjust the spatial distribution of the impinging XeF_2_ precursor molecules flux, the GIS position was changed with respect to the sample location to maximize the SIMS signal of isotopes of interest, as described in ref.^52^

### Fabrication of Li/LLZO/Li Symmetrical Cells

First, lithium metal disks (*d* ≈ 6 mm) were prepared from small pieces of a Li metal rod (Sigma Aldrich, 99.9%) rolled into a 100 μm thick foil. Subsequently, to fabricate the Li/LLZO/Li symmetric cells, lithium disks were isostatically pressed on both sides of an Al-LLZO pellet at a pressure of 1000 kN (equivalent to about 350 MPa) for 3 min in an inert environment using a PW 100 EH cold isostatic press (P/P/Weber).

### Electrochemical Measurements

The electrochemical measurements of symmetrical cells were conducted in an argon-filled glovebox, using the homemade setup (see ref (44) connected to a BioLogic VMP-300 multichannel workstation. Electrochemical impedance spectra were recorded with a BioLogic SAS MTZ-35 using a frequency range of 0.1 Hz–35 MHz with a sinus peak amplitude of 10 mV.

## Supporting Information Available

Further experimental details and characterization information (Figures S1–S12) (PDF)
