# Supplementary material for: Unveiling Surface Chemistry of Ultrafast-Sintered LLZO Solid-State Electrolytes for High-Performance Li-Garnet Solid-State Batteries
Source: Chem Mater. 2024 Nov 5;36(22):11254–63. doi: 10.1021/acs.chemmater.4c02351 (PMC11603608; doi:10.1021/acs.chemmater.4c02351)
Supplement: Supplementary file 1 — cm4c02351_si_001.pdf [file cm4c02351_si_001.pdf]

Supporting Information for

Unveiling Surface Chemistry of Ultrafast-Sintered LLZO Solid-State Electrolytes for High-Performance Li-Garnet Solid-State Batteries

Huanyu Zhang,[a,b‡] Matthias Klimpel,[a,b‡] Krzysztof Wieczerzak,[c] Romain Dubey,[a,b] Faruk Okur,[a,b] Johann Michler,[c] Lars P.H. Jeurgens,[d] Dmitry Chernyshov,[e] Wouter van Beek,[e] Kostiantyn V. Kravchyk,[a,b] and Maksym V. Kovalenko[a,b]**

[a]Laboratory for Thin Films and Photovoltaics, Empa – Swiss Federal Laboratories for Materials Science and Technology, Überlandstrasse 129, CH-8600 Dübendorf, Switzerland

[b]Laboratory of Inorganic Chemistry, Department of Chemistry and Applied Biosciences, ETH Zürich, Vladimir-Prelog-Weg 1, CH-8093 Zürich, Switzerland

[c]Laboratory for Mechanics of Materials & Nanostructures, Empa – Swiss Federal Laboratories for Materials Science and Technology, Feuerwerkerstrasse 39, CH-3602, CH-8093 Zürich, Switzerland

[d]Laboratory for Joining Technologies & Corrosion, Empa – Swiss Federal Laboratories for Materials Science and Technology, Überlandstrasse 129, CH-8600 Dübendorf, Switzerland

[e]Swiss-Norwegian Beamlines, European Synchrotron Radiation Facility, 71 Av. des Martyrs, 38000 Grenoble, France

‡These authors contributed equally.

Corresponding Authors

*E-mails: mvkovalenko@ethz.ch and Kostiantyn.Kravchyk@empa.ch

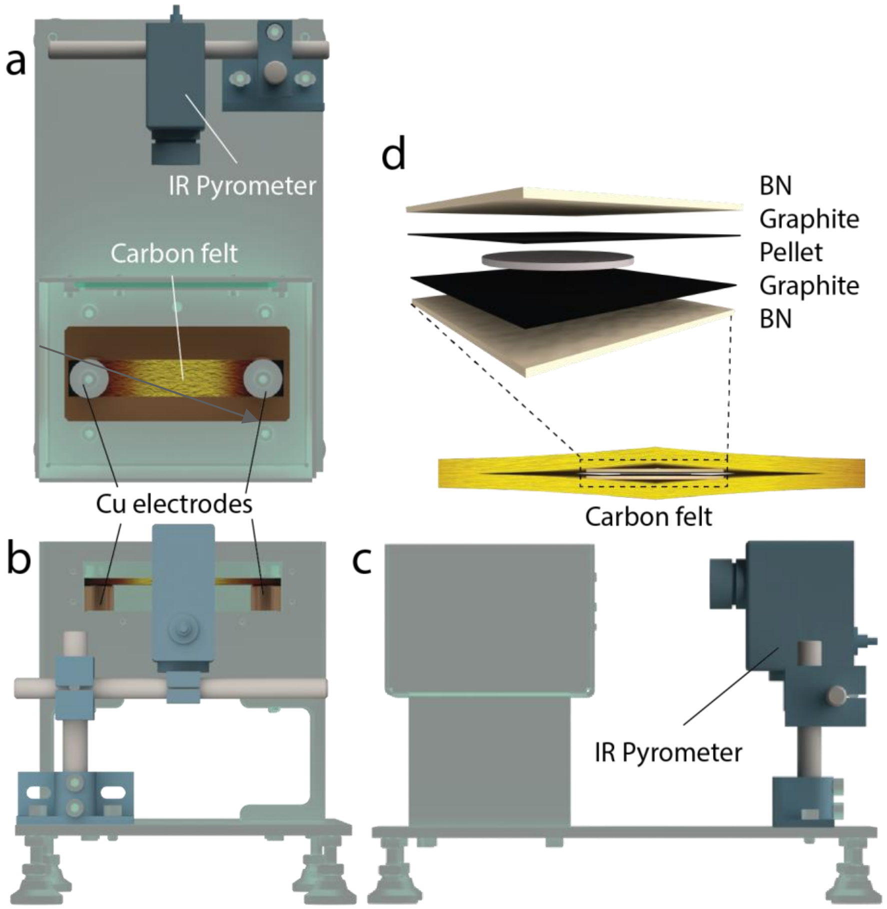

Figure S1. 3D drawings of the UFS setup from top (a) and side (b, c) views. (d) Detailed configuration of the UFS heating zone.

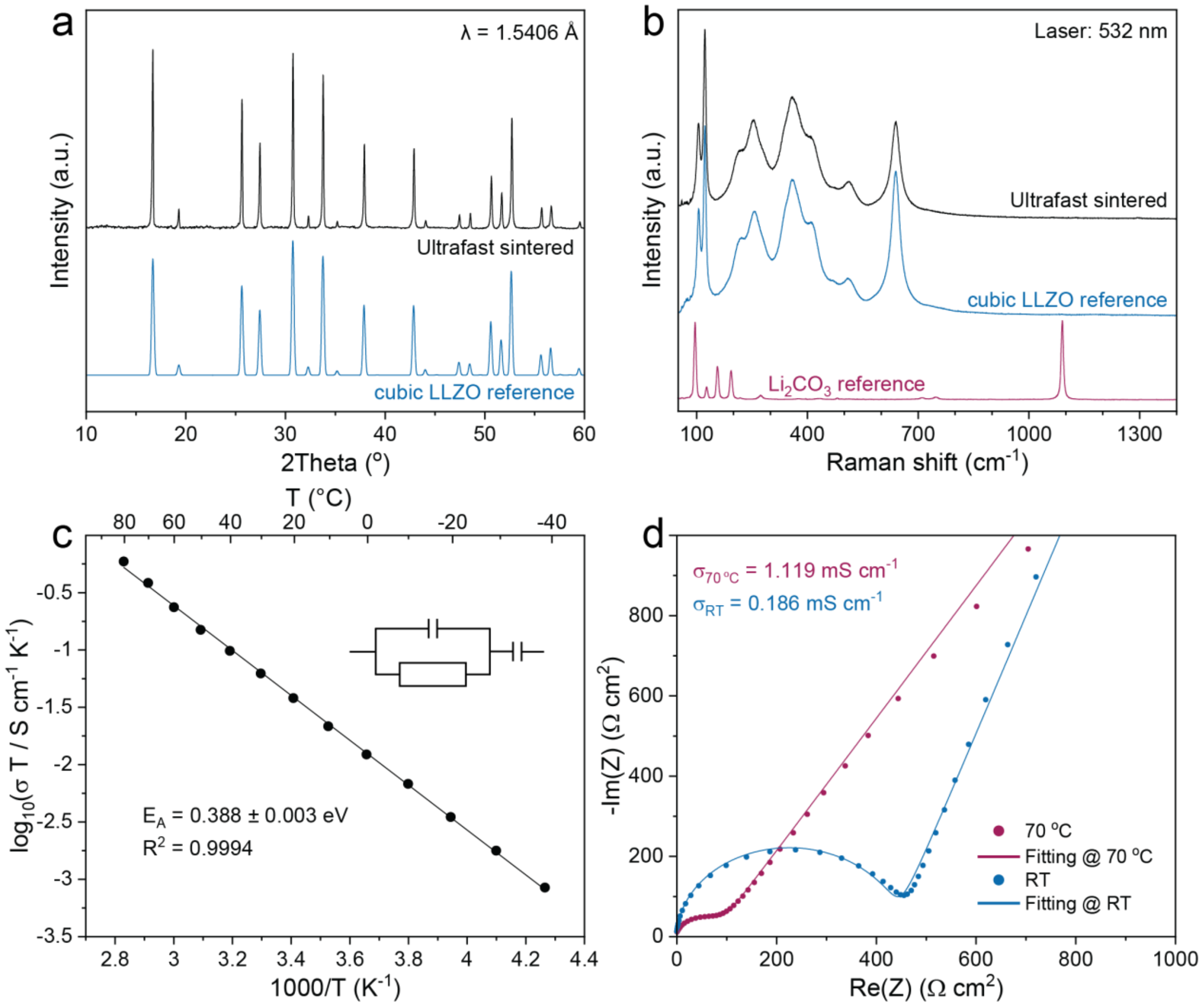

Figure S2. (a) Powder X-ray diffraction pattern and (b) Raman spectrum of as-UF-sintered LLZO pellet. X-ray diffraction pattern of cubic-LLZO structure and Raman spectra of cubic-LLZO and Li_2CO_3 are shown for comparison. (c) Arrhenius plot and (d) selected (measured at RT and 70 °C) impedance spectra (points) with fitted data (lines) of post thermal treated LLZO pellet with 50 nm Au coated on both side [inset in (c): the equivalent circuit used for fitting].

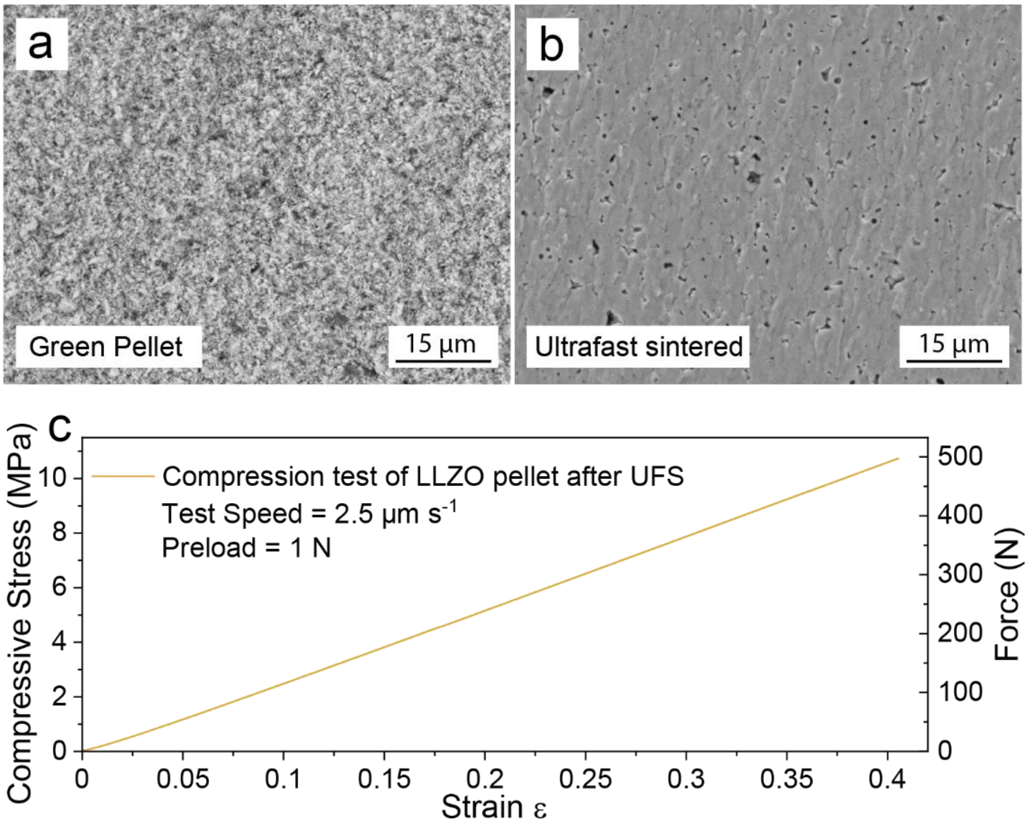

Figure S3. SEM images of LLZO pellet (a) before and (b) after UF-sintering. (c) Compression test of the UF-sintered LLZO pellet, performed at a constant piston speed of 2.5 µm s^{-1} and a preload force of 1 N.

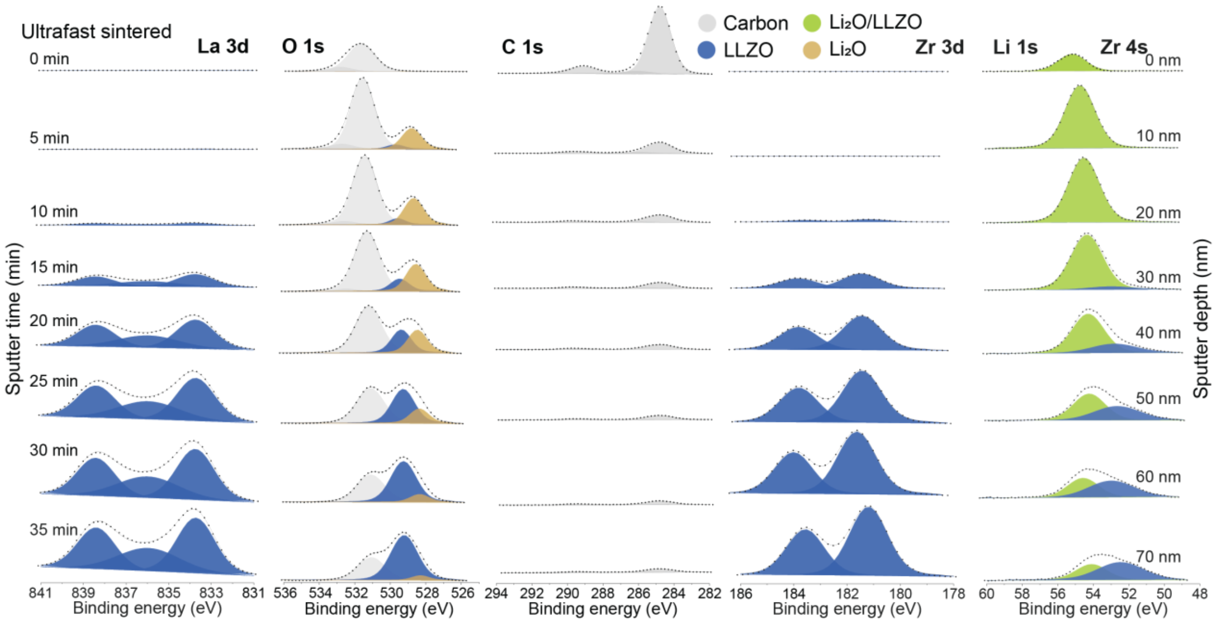

Figure S4. Charge-corrected La 3d, O 1s, C 1s, Zr 3d, Li 1s, and Zr 4s spectra of UF-sintered LLZO pellets collected on the as-sintered LLZO surface and after its sputtering for 5 to 35 min (sputtering depth of 10-70 nm).

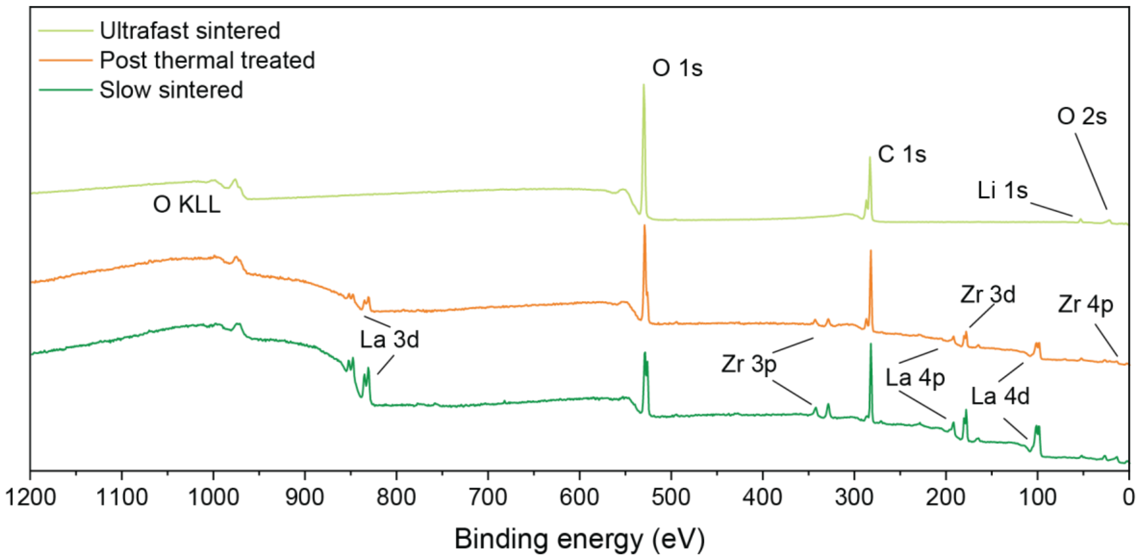

Figure S5. As measured (non-charge-corrected) XPS survey spectra of UF-sintered LLZO pellet without (green curve)/with (organge curve) heat-treatment step (900 °C, 30 min) and S-sintered LLZO pellet (dark green curve).

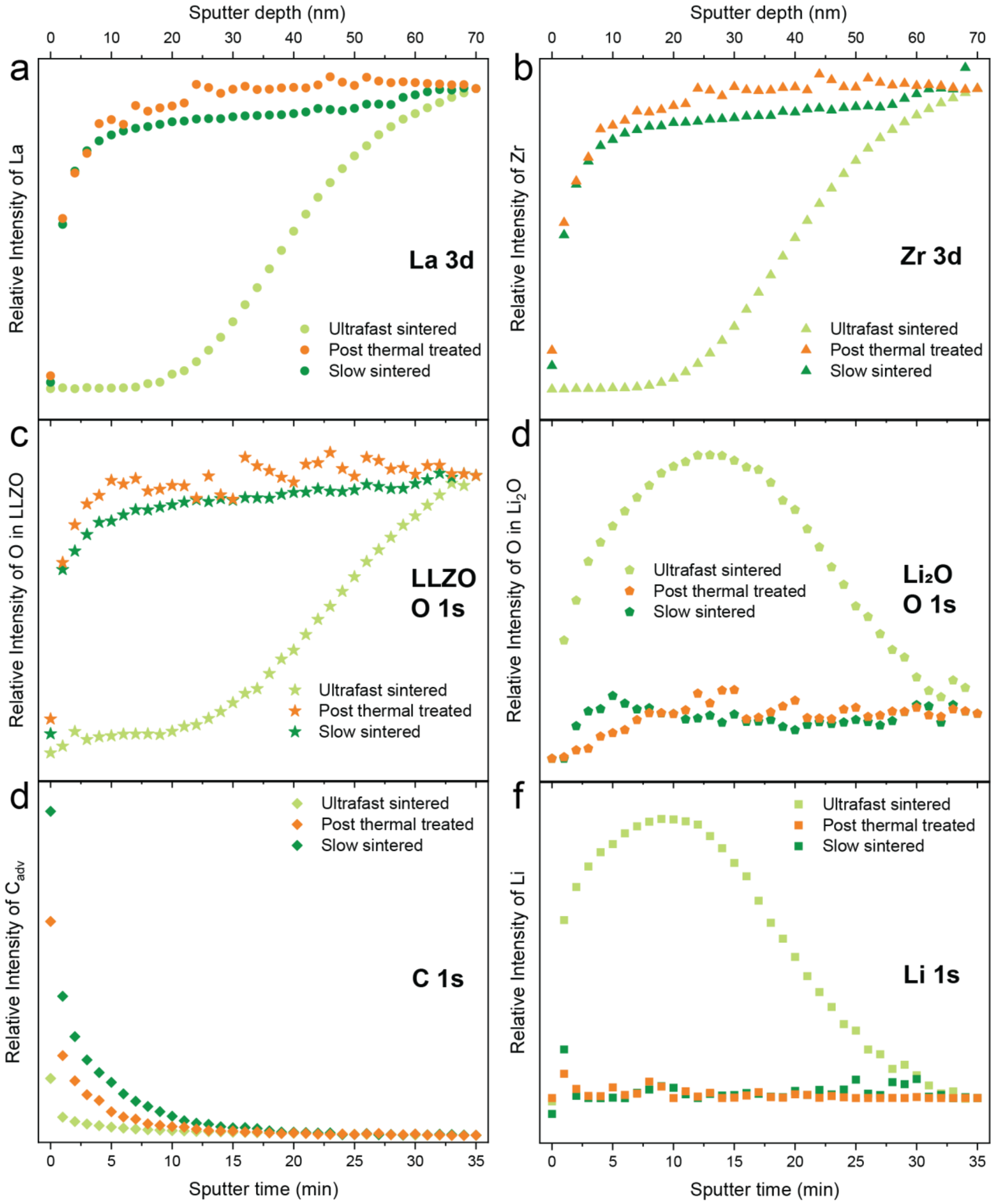

Figure S6. Depth profiles of UF-sintered LLZO pellet without (green curve)/with (blue curve) heat-treatment step (900 °C, 30 min) and S-sintered LLZO pellet (orange curve). Intensities of (a) La 3d, (b) Zr 3d, (c) O 1s (LLZO), (**d**) O 1s (Li_2O), (**e**) C 1s, and (**f**) Li1s peaks as a function of sputtering time/depth.

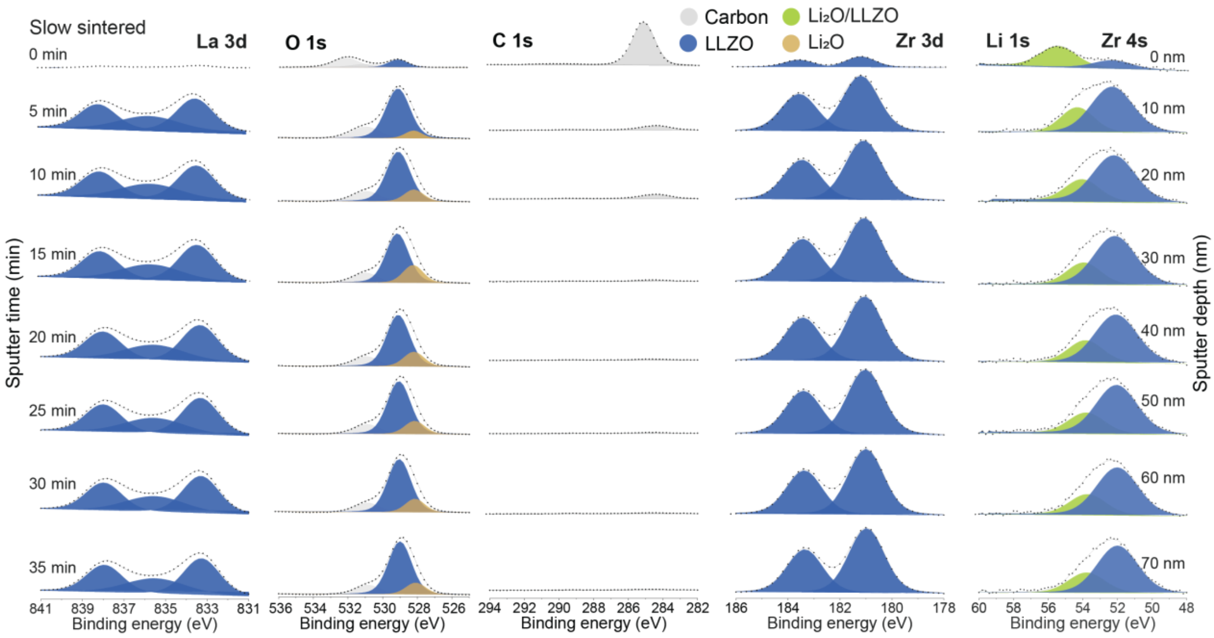

Figure S7. Charge-corrected La 3d, O 1s, C 1s, Zr 3d, Li 1s, and Zr 4s spectra of S-sintered LLZO pellets collected on the as-sintered LLZO surface and after its sputtering for 5 to 35 min (sputtering depth of 10-70 nm).

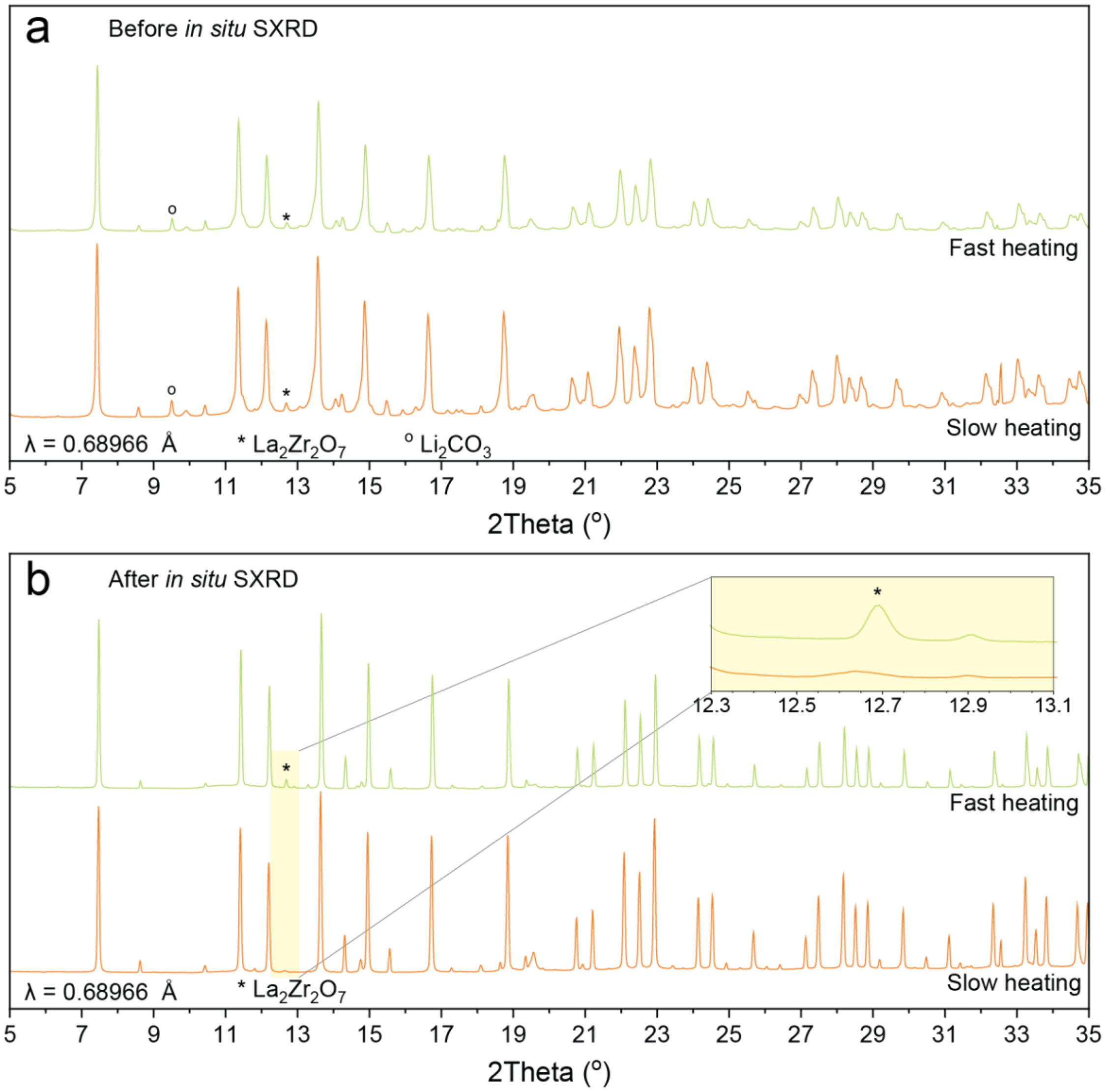

Figure S8. SXRD patterns of LLZO powder measured before (a) and after (b) heat-treatment at 1000 °C for 15 min using ultrafast (100 K min^{-1}) and slow (20 K min^{-1}) heating rates (see temperature profiles in Figures 3a and 3b). The SXRD patterns recorded during the entire heat-treatment protocol are shown in Figure 3.

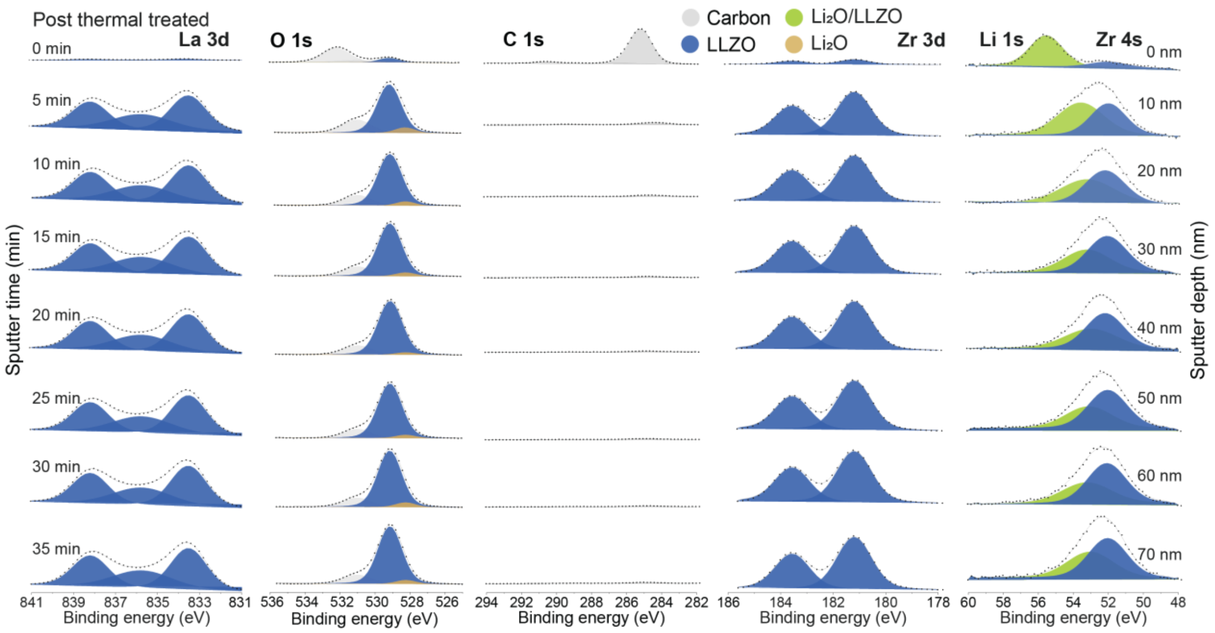

Figure S9. Charge-corrected La 3d, O 1s, C 1s, Zr 3d, Li 1s, and Zr 4s spectra of UF-sintered LLZO pellets after a heat-treatment step (900 °C, 30 min) collected on the bare LLZO surface and after its sputtering for 5 to 35 min (sputtering depth of 10-70 nm).

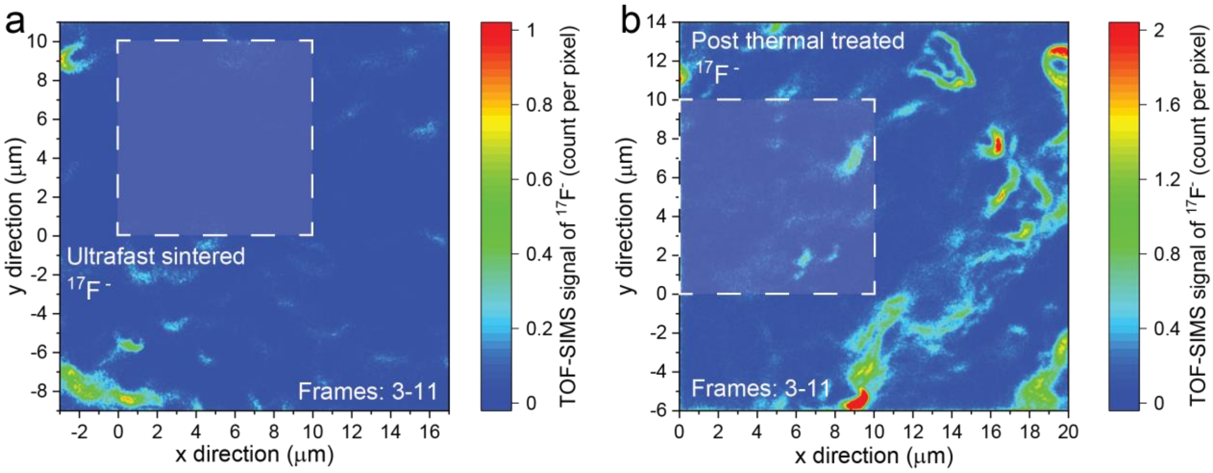

Figure S10. TOF-SIMS signal of the $^{19}F^{-}$ ion of as UF-sintered LLZO without (a) and with (b) additional 900 °C-heat-treatment step. Signal intensities are accumulated from frames 3 to 11, corresponding to depths of 100 to 500 nm.

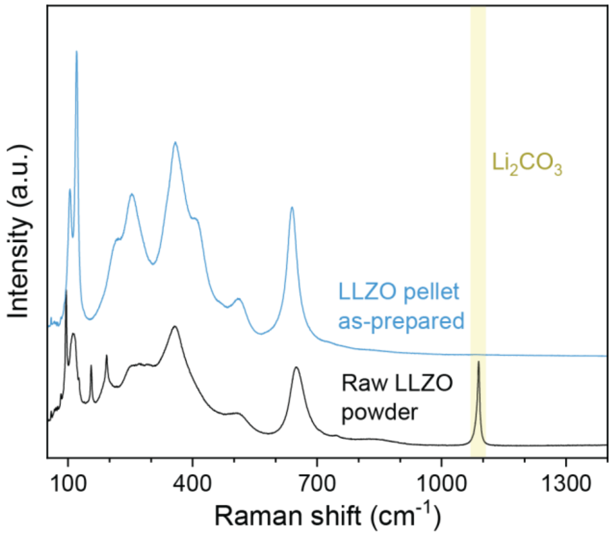

Figure S11. Raman spectra of raw LLZO powder and green body LLZO pellet after heat-treatment step at 900 ºC.

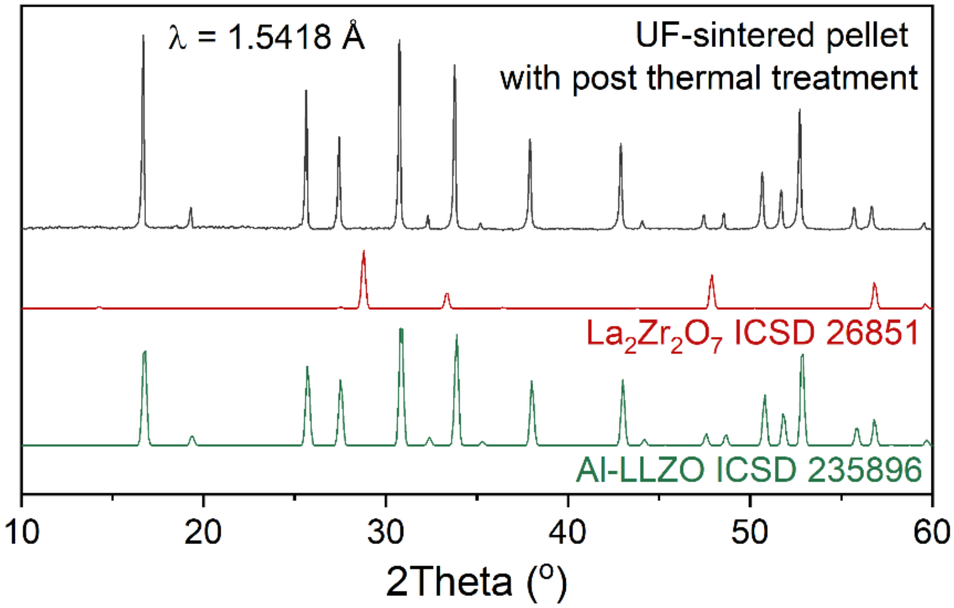

Figure S12. Powder XRD pattern of UF-sintered LLZO pellet after 900 °C-heat-treatment step.
